# Preliminary Exploration on the Regulatory Mechanism of Ionic Strength on Conformation and Hydration of Silver Carp Myosin

**DOI:** 10.3390/foods14101790

**Published:** 2025-05-18

**Authors:** Kaiqi Li, Ramy M. Khoder, Yanlei Gao, Juan You, Tao Yin, Ru Liu

**Affiliations:** 1 College of Food Science and Technology, Huazhong Agricultural University, Wuhan 430070, China; 18822057473@163.com (K.L.); ramy_khodir@fagr.bu.edu.eg (R.M.K.); gyl19970925@163.com (Y.G.); juanyou@mail.hzau.edu.cn (J.Y.); yintao@mail.hzau.edu.cn (T.Y.); 2 Engineering Research Center, Green Development, Conventional Aquatic Biological Industry, the Yangtze River Economic Belt, Ministry of Education, Wuhan 430070, China; 3 National R & D Branch Center for Conventional Freshwater Fish Processing (Wuhan), Wuhan 430070, China; 4 Faculty of Agriculture, Benha University, Moshtohor, Toukh 13736, Egypt

**Keywords:** myosin, NaCl concentration, solubility, conformation, hydration layer

## Abstract

Myosin is abundant in fish muscle tissue and plays a crucial role in gel quality of fish products. The gel forming ability of myosin is related to its solubility and conformation in ionic solution. This study investigated the regulation of NaCl concentrations (0.0~1.0 mol/L) on the solubility of silver carp (*Hypophthalmichthys molitrix*) myosin from the perspectives of conformation and hydration behavior. Results revealed that proper ionic strength (0.3~0.8 mol/L) significantly improved the solubility of myosin and reduced the average protein size (*p* < 0.05). Atomic force microscopy (AFM) observation also confirmed a decrease in the size of myosin aggregates. Increasing ionic strength induced the extending of the myosin structure and exposure of aromatic residues. These conformational changes enhanced protein–water interactions through hydrogen bonds, manifested as the formation of hydration layers. Molecular dynamics (MD) simulations also confirmed that appropriate ionic strength increased the number of hydrogen bonds between myosin and water molecules. In conclusion, proper ionic strength (0.3~0.8 mol/L)-induced exposure of polar groups in myosin enhances its hydration capacity, thereby improving solubility.

## 1. Introduction

Surimi gel products are formed by mixing fish with salt thoroughly and heating it. They are favored by consumers owing to their high nutritional value and convenience [1]. Myosin is the predominant functional protein in muscle, comprising two heavy chains (MHC) and four light chains (LC), and its gelation behavior is a critical determinant of surimi quality [2]. During heating, myosin undergoes structural transitions to form a continuous three-dimensional network via head-to-tail aggregation and disulfide crosslinking, which governs the surimi gel’s elasticity and water holding capacity (WHC) [3]. The gel formation ability of myosin is related to its solubility and conformation before heating, which can be altered by various factors, including ionic strength. Under low ionic strength, myosin readily assembles into thick filaments through intermolecular ionic bonds, leading to a significant decrease in solubility [4,5]. As the ionic strength increases, Na^+^ and Cl^−^ ions shield these electrostatic interactions, progressively disrupting the thick filament network and promoting the dispersion of myosin into monomeric units. This transition is accompanied by an elongation of the spatial conformation and an increase in α-helix content, thereby improving the solubility and gelation properties of surimi products [6].

Extensive research has established that protein hydration is fundamental to its intricate structure, stability, dynamic behavior, and physiological function [7,8]. The hydrated layer, which typically forms a complex network stabilized by hydrogen bonds, electrostatic interactions, and van der Waals forces, plays a crucial role in maintaining protein conformational stability [9]. The hydration layer of myosin comprises three distinct water populations: (1) strongly bound water directly associated with polar groups via hydrogen bonds, (2) weakly bound water regulated by hydrophobic interactions, (3) free water influenced by electrostatic shielding effects [10]. Ionic strength modulates this system through dual synergistic mechanisms: Na^+^ not only attenuate intermolecular electrostatic interactions through charge screening effects [11] but also competitively displace hydration water molecules from protein surface binding sites, thereby inducing structural reorganization of the hydration layer [12]. Additionally, studies have shown that increased ionic strength significantly weakens the electrostatic interactions in bovine serum albumin, which impedes protein chain folding and reduces protein aggregation [13]. Xiong et al. [14] noted that ethanol disrupts the hydration layer of myosin, leading to aggregation. Consequently, augmenting the thickness of the protein hydration layer and water molecular density can effectively enhance protein conformational stability [15,16]. Although current studies have established macroscopic correlations between ionic strength and solubility variations, the mechanism remains elusive on hydration behavior mediating structural stability and solubility of myosin.

This study investigated the impact of varying ionic strengths on myosin’s solubility and spatial structure. Subsequently, the dynamic alterations in the hydration layer were quantitatively assessed using bio-layer interferometry (BLI), isothermal titration calorimetry (ITC) and melting phase transition analysis. Finally, molecular dynamics simulations were employed to further clarify the mechanism on hydration behavior mediating structural stability and solubility of myosin. This study offers theoretical guidance for enhancing gel texture and water holding capacity, under different ionic strength, thereby supporting the development of healthier and higher gel quality fish products.

## 2. Materials and Methods

### 2.1. Materials

All standard animal handling protocols strictly complied with the institutional guidelines established by Huazhong Agricultural University’s Animal Ethics Committee (Approval ID: HZAUFI-2023-0027). Fresh live silver carp (*Hypophthalmichthys molitrix*) were procured from a supermarket at Huazhong Agricultural University in Wuhan, Hubei, China.

Sodium chloride (NaCl), tris(hydroxymethyl)-aminomethane (Tris), β-mercaptoethanol (β-ME), adenosine disodium triphosphate (ATP-Na_2_), and other chemical reagents were of analytical grade and were provided by Sinophasic Chemical Reagent Co., Ltd. (Shanghai, China).

### 2.2. Myosin Extraction

Myosin was extracted according to the method of Gao et al. [17] with minor modifications. Buffer A (0.1 mol/L KCl, 20 mmol/L Tris-HCl, pH 7.5) and buffer B (0.45 mol/L KCl, 5 mmol/L β-ME, 0.2 mol/L Mg(CH_3_COO)_2_, 1 mmol/L EGTA, 20 mmol/L Tris-HCl, pH 6.8), and buffer C (0.5 mol/L KCl, 5 mmol/L β-ME, 20 mmol/L Tris-HCl, pH 7.5) were prepared in advance and stored at 4 °C until use.

**Figure 1:**
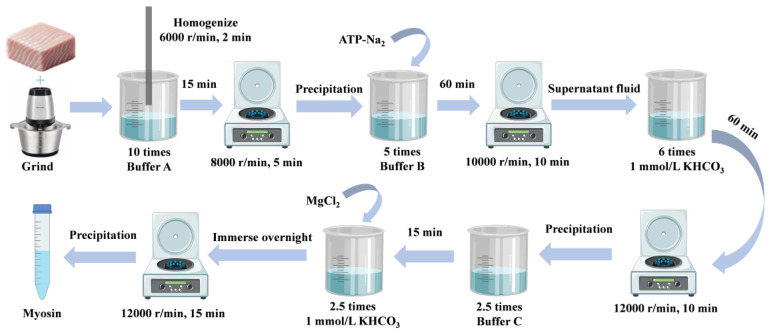
Myosin extraction process.

The silver carp were promptly transported alive to the laboratory. They were immediately headed, gutted, and washed with water. The dorsal muscle was separated and ground using a conditioning machine. Subsequently, 10 times the volume of buffer A was added to the ground meat, and the mixture was homogenized at 6000 rpm for 2 min with a high-speed dispersion homogenizer. The homogenized suspension was then incubated at 4 °C for 15 min, after which the supernatant was removed following centrifugation at 8000 rpm for 5 min. The precipitate was suspended in a 5-fold volume of buffer B, and ATP-Na_2_ was added to dissociate myosin from actin, achieving a final concentration of 5 mmol/L. This mixture was placed at 4 °C for 60 min and centrifuged at 10,000 rpm for 10 min. The supernatant was diluted with a 6-fold volume of 1 mmol/L KHCO_3_ solution, incubated at 4 °C for 60 min, and then centrifuged at 12,000 rpm for 10 min. The precipitate was resuspended in 2.5 times the volume of buffer C, incubated at 4 °C for 15 min, and then diluted with 2.5 times the volume of 1 mmol/L KHCO_3_ solution. Solid MgCl_2_ was added to achieve a final concentration of 10 mmol/L, and the mixture was placed at 4 °C overnight. Finally, the precipitate was centrifuged at 12,000 rpm for 15 min to obtain purified myosin, with all operational steps conducted at 4 °C (Figure 1).

The extracted myosin was adjusted to different ionic strengths (0.0, 0.1, 0.2, 0.3, 0.4, 0.5, 0.6, 0.8, 1.0 mol/L) with 20 mmol/L Tris-HCl buffer (pH 7.5, containing various concentrations of NaCl), and the concentration of protein was controlled to 5 mg/mL. The changes in each index were measured by placing 25 mL of myosin suspension into 50 mL centrifuge tube.

### 2.3. Determination of Solubility

After various treatments, the myosin suspension was centrifuged at high speed (8000 r/min, 15 min, 4 °C). The protein content in the supernatant was quantified using the Lowry’s method [18] using bovine serum albumin (BSA) as the standard. Myosin solubility was calculated using the following formula:

(1) $$Solubility\left(\%\right)=\frac{C_{1}}{C_{2}}\times 100$$

*C*_1_: The protein concentration of the myosin supernatant following centrifugation;

*C*_2_: The protein concentration of myosin suspension was 5 mg/mL.

This procedure was repeated three times to obtain an average value.

### 2.4. Determination of Turbidity

Turbidity was determined as described by Gao et al. [17]. The concentration of myosin suspension was adjusted to 1.0 mg/mL and allowed to equilibrate at room temperature for 30 min. The turbidity was then measured using an ultraviolet spectrophotometer at 320 nm.

### 2.5. Determination of Particle Size

The particle size was measured using a Malvern laser particle size analyzer (Mastersizer 3000, Malvern Instruments Ltd., Malvern, UK) based on dynamic light scattering principles [19]. The measurement conditions included a water solvent with a viscosity of 0.8873 mPa·s, a medium refractive index of 1.33, and a material refractive index of 1.45. The relevant measurement time function was analyzed by an automatic program, which subsequently outputs the average protein size via computer. For the analysis, 2 mL of sample suspension was aspirated into a “polystyrene” sample cell and placed into the instrument for testing at 25 °C (laser wavelength: 633 nm, scattering angle: 173°). Each sample underwent three tests, incorporating laser scanning for 15 cycles.

### 2.6. Determination of Zeta Potential

The Zeta potential of myosin samples was assessed using a Malvern laser particle size analyzer (Mastersizer 3000, Malvern Instruments Ltd., Malvern, UK) equipped with a Laser Doppler Electrophoresis detector according to the method of Zhang et al. [20]. The measurements were conducted at 25 °C, with a temperature balance time of 2 min. Each group of measurements was repeated more than three times to ensure accuracy.

### 2.7. Observation of Microstructure

The microstructure of myosin was observed using atomic force microscopy (AFM) according to the method of Xiong et al. [14]. The concentration of myosin samples was adjusted to 20 μg/mL with 20 mmol Tris-HCl buffer. A total of 10 μL of the treated myosin suspension was dropped on the newly stripped mica sheet. To prevent dust pollution in the air, the mica sheet with samples was placed on an ultra-clean table and dried naturally at room temperature. The dried myosin would be adsorbed on the surface of the mica sheet. The air-dried samples were rinsed five times with 35 μL of ultrapure water to remove the salts. After the samples were allowed to air dry again naturally, the aggregation state of myosin was observed by AFM (Multimode 8, Bruke Co., Billerica, MA, USA). TAP-150 microprobe was selected, and a frequency swept operation was performed using molecular force mode. The microcantilever thickness of the AFM was 2 μm, the resonance scanning frequency was selected to be 180 kHz, and the resolution of the image was set to 256 × 256. The probe was 115 μm to 135 μm in length, 25 μm to 35 μm in width, 5 N/m in elasticity, and 5 μm × 5 μm in scanning area. The corresponding images were analyzed by Nanoscope Analysis 1.8 software.

### 2.8. Determination of the Secondary Structure

A J-1500 circular dichroism spectrometer determined the circular dichroism of myosin in different treatment groups. The samples with a concentration of 0.01 mg/mL were placed into a quartz sample cell with an optical diameter of 0.1 cm and scanned in the far-UV region (190~250 nm) at a scanning rate of 50 nm/min with a response time of 5 s. Each sample was conducted in triplicate and averaged, with the average molecular weight of an amino acid residues taken as 110 g/mol. The Young’s method was employed to calculate the α-helix, β-sheet, β-turn, and random coil content of myosin [21]. Characteristic spectral minima were identified as follows: α-helix at 208 nm and 222 nm; β-sheet at 216 nm; β-turn between 220~230 nm; and random coil at 200 nm.

### 2.9. Determination of the Tertiary Structure

#### 2.9.1. Determination of UV Absorption Spectra and Second Derivatives

The UV absorption spectra were determined following the Gao et al. [22] with minor modifications. The concentration of the sample was adjusted to 1.0 mg/mL, and 20 mmol/L Tris-HCl buffer (pH 7.5) with the appropriate NaCl concentration served as the reference blank. A UV-1750 UV-visible spectrophotometer was used to scan at medium speed in the 230 to 350 nm range with a sampling interval of 1 nm. The spectral data were subjected to second-order derivative transformation using the Differentiate mode in Origin 8.0 software (Origin Lab, Northampton, MA, USA). To minimize noise interference, baseline correction and smoothing procedures (9-point window, second-order polynomial) were applied through the software prior to second-order derivative analysis.

#### 2.9.2. Determination of Fluorescence Spectra

Changes in fluorescence spectra provide indirect insights into protein conformational changes. Fluorescence spectra were determined as described by Yu et al. [23], with some modifications. The protein suspension of the various treatments were diluted to a protein concentration of 0.5 mg/mL and then determined using a fluorometer. The determination conditions were as follows: the excitation wavelength was 295 nm, the slits were all 5 nm, the scanning range was 300 to 450 nm, and the sensitivity was 2.

### 2.10. Determination of Surface Hydrophobicity (S_0_-ANS)

According to the method of Yongsawatdigul and Sinsuwan [24] with minor modifications, 8-aniline-1-naphthalenesulfonic acid (ANS) was used as a fluorescent probe to measure the surface hydrophobicity, and the myosin concentration was adjusted to 0.05, 0.10, 0.15, and 0.20 mg/mL, respectively. First, 20 μL of 8 mmol/L ANS-0.1 mol/L potassium phosphate buffer (pH 7.4) was thoroughly mixed into 4 mL of the sample. The corresponding buffer solution was used as a blank. A fluorescence spectrometer (model F-4600, Hitachi, Tokyo, Japan) was used to determine the fluorescence intensity of the samples at an excitation wavelength of 390 nm (slit 2.5 nm) and an emission wavelength of 470 nm (slit 2.5 nm).

### 2.11. Determination of Hydration Behavior

#### 2.11.1. Determination of Thermal Changes in the Interaction of Myosin with NaCl

ITC was conducted following the methodology of Xiong et al. [25]. About 350 μL of the myosin suspension was added to the sample cell. The injection needle drew 50 μL of titration solution (3.0 mol/L NaCl, pH 7.5). Constant temperature stirring was set, with the stirring rate at 250 rpm. The system was then used to infuse the predetermined guest solution at a constant, pre-set feeding rate. The calorific values of the mixed system in the reaction vessel were measured after 10 min equilibration period or upon stabilization of the temperature data. Calorific values were recorded once the temperature data reached equilibrium. Measurements were replicated a minimum of three times for each experimental group.

#### 2.11.2. Determination of the Interaction of Myosin with NaCl

BLI was employed to quantify the interaction between NaCl and myosin. Purified myosin in Tris–HCl buffer, was immobilized onto Super Streptavidin (SSA) biosensors of a real-time, label-free molecular interaction system (Octet, ForteBio, Fremont, CA, USA). NaCl solutions in the same buffer, were loaded into a 96-well plate. The myosin-coated biosensors were first immersed in NaCl solution for a 120 s association phase, then transferred to buffer-only wells for a 120 s dissociation phase. Binding and dissociation data were recorded in real time using the Octet Data Acquisition 11.1.0.11 software and fitted to a 1:1 binding model to calculate the association rate constant (k_on_), dissociation rate constant (k_off_), and equilibrium dissociation constant (KD). These kinetic parameters were subsequently used to characterize the binding affinity of NaCl for myosin.

#### 2.11.3. Determination of Thermal Characteristics of Melting Phase Transition

The melting thermal properties of myosin were determined by the DSC method described by Zhang et al. [26]. Approximately 10 mg of the sample was precisely weighed into an aluminum crucible and hermetically sealed using a press. The sealed sample was then loaded into the DSC instrument, and the temperature program was set to scan from −25 °C to 10 °C at a heating rate of 1 °C/min. Measurements were performed using a liquid nitrogen cooling system (Q2000, TA Instruments, New Castle, DE, USA). The following parameters were obtained by analyzing the heat-flow curve: Onset temperature (T_o_): Temperature at which the endothermic signal initially deviates from the baseline. Peak temperature (T_p_): Temperature corresponding to the maximum melting rate (i.e., the apex of the endothermic peak). End temperature (T_e_): The temperature at which the signal stabilizes to the baseline. The enthalpy change (ΔH) was calculated by integrating the area under the endothermic peak, representing the total heat absorbed during the denaturation process.

### 2.12. Molecular Dynamics Simulation (MD)

The amino acid sequence of the silver carp myosin heavy chain (ID: A8R0Q2) was modeled and optimized in the “Build Ontology Models” module, using structure 5TBY for multi-template homology modeling to obtain the spatial structure of myosin domain. The simulation box was filled with NaCl solutions at 0.1 mol/L and 0.5 mol/L, respectively. The OPLS-AA all-atomic force field was selected, and the SPC/E water molecule model was used. The Berendsen method was employed to control temperature and pressure, with the pressure set to 1.01 × 10^5^ Pa and the pressure coupling constant at 2.0 ps. The LINCS algorithm was used to constrain intramolecular bond interactions. The non-bonded van der Waals interactions were calculated using the Lenard-Jones potential with a cutoff distance of 1.0 nm. The Particle-Grid Ewald method was utilized to handle electrostatic interactions.

The steepest descent method was initially applied to minimize the system’s energy and remove inappropriate positions. Subsequently, 2 nanosecond (ns) NVT (constant Number of particles, Volume, and Temperature) ensemble simulation was executed, followed by 2 ns NPT (constant Number of particles, Pressure, and Temperature) ensemble simulation to achieve equilibrium. In the NPT ensemble of each system, a 20 ns molecular dynamics (MD) simulation was run in 20 femtosecond (fs) steps. NPT integration was performed in each system, and the last 19 ns of data was collected at 10 picosecond (ps) saving frequency for subsequent analysis. The MD simulations were conducted using GROMACS 5.0 software, with a total simulation time of 20 ns.

### 2.13. Statistical Analysis

All experiments were conducted in triplicate, with a minimum of three replicates for each trial. The experimental data were analyzed using SPSS 26.0 (IBM SPSS Institute Inc., Chicago, IL, USA). Triplicate measurements were analyzed via one-way ANOVA with Dunnett’s post hoc test, and a *p*-value of less than 0.05 was deemed statistically significant. All charts were created using Origin 2021 (Origin Lab, Northampton, MA, USA).

## 3. Results

### 3.1. Solubility

**Figure 2:**
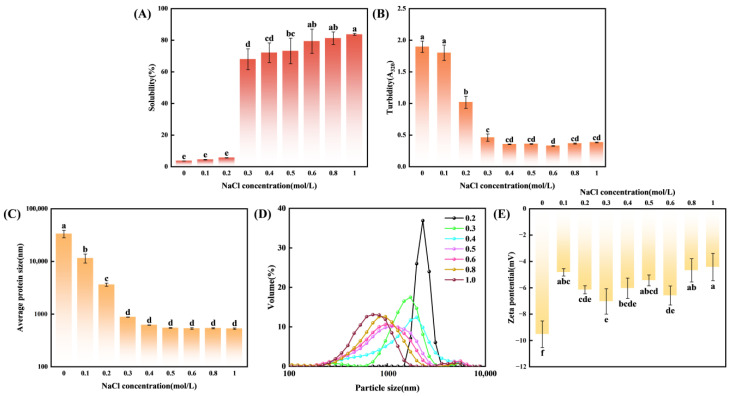
Effect of ionic strength on the physicochemical properties of myosin. (**A**) solubility; (**B**) turbidity; (**C**) average protein size; (**D**) particle size; (**E**) zeta potential. Different lowercase letters indicate significant difference between different ionic strengths (*p* < 0.05).

The solubility of myosin under varying ionic strengths is shown in Figure 2. Myosin solubility at ionic strengths ranging from 0.3 to 1.0 mol/L was significantly higher than that at 0.0~0.2 mol/L (*p* < 0.05). As ionic strength increased from 0.3 mol/L to 0.6 mol/L, myosin solubility exhibited an upward trend, with no significant difference after exceeding 0.6 mol/L, while turbidity displayed an inverse relationship (Figure 2A,B). This phenomenon may be attributed to myosin aggregation into insoluble thick filaments under low ionic strength conditions. Elevating ionic strength promoted the disintegration of thick myofilaments, thereby improving the solubility of myosin [27,28]. Similarly, Xue et al. [29] observed myosin aggregates as filaments at low ionic strength (0.2 mol/L). Notably, suspension turbidity exhibited a slight increase at higher ionic strengths (0.8~1.0 mol/L). This reversal may have resulted from the salting-out effect caused by excessive ionic strength, which led to the partial reaggregation of protein molecules [30].

The particle size served as an indicator of the degree of protein aggregation [31]. At low ionic strength (0.0~0.1 mol/L), myosin formed large aggregates (>10 µm; Figure 2C). Raising the ionic strength to 0.2 mol/L significantly reduced the average protein size (*p* < 0.05). As shown in Figure 2D, the size distribution exhibited a single peak between 1800 and 3600 nm. As the ionic strength increases, the peak gradually shifts to the left, reflecting the de-composition of large aggregates and more homogeneously dispersed particles, which was consistent with enhanced solubility. Beyond 0.6 mol/L, the average protein size tended to stabilize (Figure 2C), which was in line with the trends in solubility and turbidity (Figure 2A,B). At low ionic strength, rod–rod ionic bonds droved the formation of myosin filaments and large aggregates. Charge shielding by salt ions disrupted these bonds, promoting dissociation into oligomers or monomers and thereby improving solubility [32].

As depicted in Figure 2E, the zeta potential of all samples exhibited negative values, indicating that the protein possessed a net negative charge. Adding salt ions significantly decreased the absolute value of the zeta potential. This originated from interactions between Na^+^ ions and the carboxyl groups on the myosin surface, which neutralized the protein’s negative charges [33,34,35,36]. With further increases in ionic strength, the zeta potential of myosin slightly increased (*p* > 0.05).

### 3.2. Microorphology

**Figure 3:**
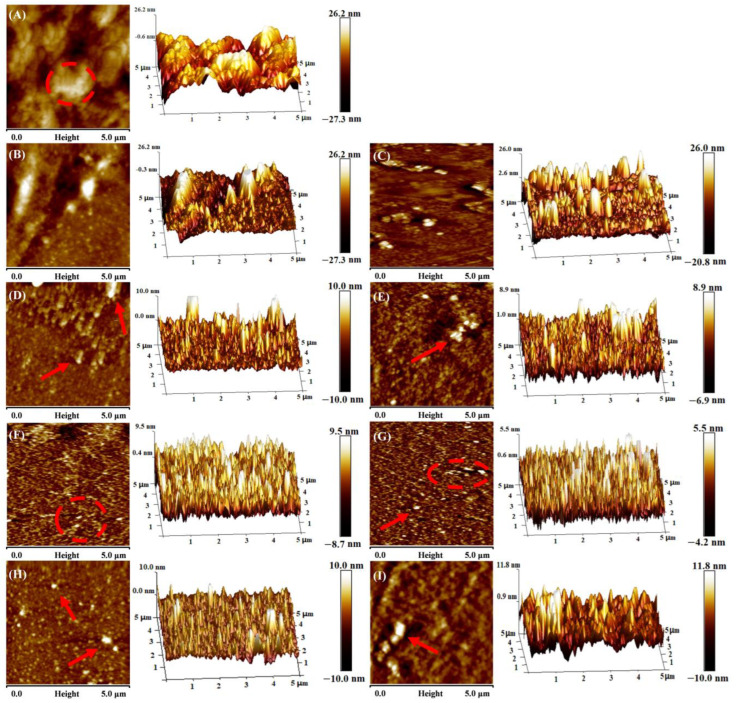
Effect of ionic strength on the micromorphology of myosin. (**A**) 0.0 mol/L; (**B**) 0.1 mol/L; (**C**) 0.2 mol/L; (**D**) 0.3 mol/L; (**E**) 0.4 mol/L; (**F**) 0.5 mol/L; (**G**) 0.6 mol/L; (**H**) 0.8 mol/L; (**I**) 1.0 mol/L. The red arrows indicated protein particles. The red circles indicated the aggregation degree of protein particles.

Figure 3 displayed the micromorphology of myosin under varying ionic strength conditions. At an ionic strength of 0.0 mol/L, the diameter of myosin aggregates reached 2 μm. The distribution was uneven, and the aggregate height attained 26.2 nm, suggesting that myosin aggregated or overlapped under these conditions. As ionic strength increased, the size of aggregates gradually diminished, the distribution became more uniform, and the height decreased to 5.5 nm (0.6 mol/L). Similarly, Epstein et al. [37] reported that the diameter of myosin filaments gradually decreased from 33.4 nm to 15.2 nm with increasing KCl concentration from 0.25 mol/L to 0.45 mol/L. However, when the ionic strength became excessively high (0.8 to 1.0 mol/L), myosin aggregation reappeared, exhibiting irregular shapes and a slight increase in height. This may be attributed to the salting-out effect, where myosin molecules re-agglomerate to form larger aggregates.

### 3.3. Spatial Structure

#### 3.3.1. Secondary Structure

**Figure 4:**
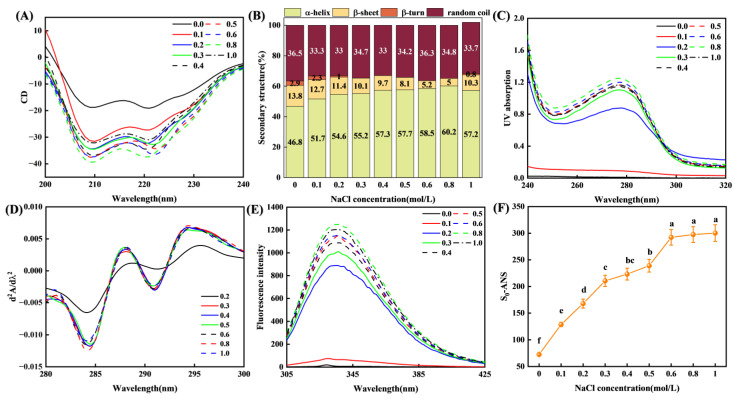
Effect of ionic strength on the spatial structure of myosin. (**A**) CD spectrum; (**B**) secondary structure; (**C**) ultraviolet absorption spectrum; (**D**) second-order conductance spectrum; (**E**) fluorescence spectrum; (**F**) surface hydrophobicity. Different lowercase letters indicate significant difference between different ionic strengths (*p* < 0.05).

As shown in Figure 4A, the CD spectra of myosin under different ionic strengths exhibited two prominent peaks near 208 nm and 222 nm, suggesting that the α-helix was the predominant secondary structure [38]. When the ionic strength increased from 0.0 to 0.8 mol/L, the α-helix content increased from 46.8% to 60.2%, accompanied by a decrease in β-sheet content from 13.8% to 5.0% (Figure 4B). It has been reported that higher ionic strength could facilitate the formation of additional hydrogen bonds between proteins, thereby stabilizing the α-helix structure [39]. However, when the ionic strength exceeded 0.8 mol/L, the α-helix content decreased to 57.2%, while the β-sheet content increased to 10.3% (Figure 4B), suggesting a loosening of the protein conformation [40,41].

#### 3.3.2. Tertiary Structure and Surface Hydrophobicity

Figure 4C depicted the ultraviolet absorption spectrum of myosin suspension across various ionic strengths. Within the 0.2 to 1.0 mol/L ionic strength range, a characteristic absorption peak corresponding to aromatic amino acids emerged in the myosin samples at 278 nm. The intensity of this peak increased with increasing ionic strength, suggesting that an elevation in ionic strength facilitated the elongation of myosin molecules, thereby exposing a greater number of aromatic amino acid residues. Conversely, at lower ionic strengths (0.0 to 0.1 mol/L), the ultraviolet absorption value of the myosin suspension was relatively low, and no characteristic absorption peak was observed. It was speculated that myosin aggregated under these conditions, leading to the embedding of aromatic amino acid residues into the aggregates. Furthermore, the ultraviolet absorption band centered around 278 nm spans a wide spectral range (250~300 nm), which was produced by the overlapping contribution of aromatic amino acids (tryptophan, tyrosine, and phenylalanine) [42]. To resolve overlapping spectral features within this broad band, second-order derivative UV spectra were analyzed (Figure 4D). The increasing intensity of the peaks at 288 nm and 298 nm with rising ionic strength indicated progressive exposure of tyrosine–tryptophan interaction sites and individual tryptophan residues to the solvent [30], once again verified the structural extending.

As illustrated in Figure 4E, myosin samples subjected to varying ionic strengths exhibited characteristic fluorescence peaks near 340 nm. As ionic strength increased, the fluorescence intensity of myosin samples gradually augmented, indicating that the protein structure may have extended [43,44]. However, the fluorescence intensity diminished when NaCl concentrations reached 1.0 mol/L. This decrease may have been a consequence of Na^+^ competing with myosin for the surrounding water molecules, leading to partial shielding of myosin’s hydrophobic residues and their rearrangement into a more compact tertiary structure [45,46,47]. Furthermore, as ionic strength increased, the surface hydrophobicity of myosin initially rose and subsequently plateaued (Figure 4F). The gradual dissociation of myosin may have resulted in partial protein extending and exposure of hydrophobic groups, thereby augmenting surface hydrophobicity [48,49]. Notably, within the ionic strength range of 0.6 to 1.0 mol/L, no significant differences were observed, which aligned with the results obtained from the fluorescence spectrum (Figure 4E).

### 3.4. Hydration Behavior

#### 3.4.1. Isothermal Titration Calorimetry (ITC)

**Figure 5:**
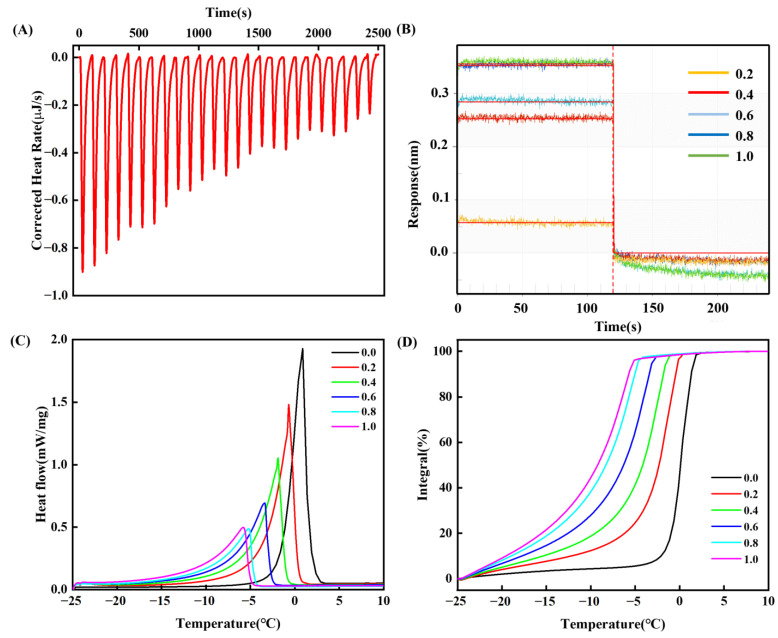
Effect of ionic strength on hydration properties of myosin. (**A**) Thermal map of the binding process of myosin and NaCl; (**B**) myosin binding dissociation curve with NaCl; (**C**) heat flow diagram of the myosin melting process; (**D**) integral value of the peak area of the myosin melting process.

ITC is a technique used to study biomolecule interaction by measuring heat exchange [50]. The isothermal titration curve for a myosin suspension titrated with NaCl is depicted in Figure 5A, and the thermodynamic parameters of this interaction are presented in Table 1. According to the isothermal titration curve, the heat exchange observed upon the gradual addition of NaCl to the myosin suspension exhibited a regular decline (Figure 5A). The enthalpy change (ΔH) and the entropy change (ΔS) were found to be greater than zero. According to Huo et al. [51], when ΔH and ΔS are positive, it suggests that the primary force driving the system is hydrophobic interaction, which was in agreement with the results obtained for surface hydrophobicity (Figure 4F). It has been reported that NaCl altered the charge distribution of myosin, enhanced its solubility, and disrupted aggregates into monomers or oligomers, thereby exposing more hydrophobic amino acid residues on myosin and intensifying the hydrophobic interactions between protein molecules [52]. The hydrophobic interaction resulted in a negative Gibbs free energy (ΔG) of −8.061 kJ/mol, indicating that the binding of NaCl to myosin occurred spontaneously [53]. Additionally, adding NaCl modified the arrangement of water molecules surrounding the protein. It attracted water molecules to form a hydrated layer due to the strong interaction between Na^+^ and Cl^−^ ions and water molecules [54]. This rearrangement of water molecules may have led to an increase in the water molecular layer on the surface of myosin (ΔS > 0). A positive ΔH also indicates an increase in the hydration layer of myosin [55].

#### 3.4.2. Bio-Layer Interferometry (BLI)

BLI enables rapid detection of binding kinetics between proteins and small molecules. As shown in Figure 5B, the binding of myosin to NaCl exhibited a pronounced concentration dependence across ionic strengths from 0.2 to 1.0 mol/L. The observed variation in sensor thickness can be attributed to three synergistic factors: ion–protein binding, conformational extension, and hydration layer thickening. When ionic strength increased from 0.2 mol/L to 0.4 mol/L, the sensor response signal showed the maximum increase (ΔR = 0.2 nm). Consistent with the solubility analysis in Figure 2A, this initial ionic strength enhancement reduced intermolecular electrostatic interactions via charge shielding, thereby improving solubility. Meanwhile, it induced conformational extension of myosin, exposing polar residues on the protein surface and promoting the formation of a structured hydration layer [14]. However, when ionic strength rose further to 0.8 mol/L, the response amplitude declined significantly, and the spatial conformation remained largely unchanged (Figure 4C). Concurrently, the number of available ion-binding sites on the protein surface decreased, and no further enhancement in sensor response was observed at ionic strengths above 0.8 mol/L. This stabilization likely resulted from intensified hydration competition between myosin surface groups and Na^+^/Cl^−^, which weakened protein–water interactions. Ultimately, the system attained dynamic equilibrium between these competing processes. After fitting and analysis, the binding equilibrium dissociation constant KD value was 6.70 × 10^−2^ M, the binding rate constant K_on_ was 2.69 × 10^2^ M^−1^S^−1^, and the dissociation rate constant k_dis_ was 18.02 S^−1^. These values indicate that the myosin–NaCl interaction is characterized by rapid association and dissociation [56], in agreement with the isothermal titration calorimetry results (Table 1).

#### 3.4.3. Thermal Characteristics of Melting Phase Transition

The thermal properties of the molten phase transition reflect the absorption of heat from the solid to the liquid state of the water in the sample during the heating process [57]. As illustrated in Figure 5C, at 0.0 mol/L ionic strength, myosin exhibited an endothermic peak temperature at 0.76 °C, representing the melting temperature of ice crystals within the myosin sample. A smaller ΔH (i.e., a reduced peak area) indicated less heat absorption during melting, reflecting a lower content of freezable water. Likewise, a shift of T_p_ to lower values and a broadening of the peak reflected stronger water–protein interactions [58]. The absorbed heat amounted to 262.97 J/g during the melting process (Table 2). The elevation of ionic strength led to the reductions in T_o_, T_p_, and T_e_ values. The ionic strength of 0.8 mol/L has the lowest T_p_ (−5.22 °C) and ΔH (110.85 J/g). This observation suggested a reduction in frozen water content in the myosin samples with increasing ionic strength, resulting from myosin conformational extension that exposed hydrophilic groups to bind free water and form a stabilized hydration layer [59]. A shift of T_p_ to lower values and a broadening of the peak reflected stronger water–protein interactions [60]. Figure 5C,D further revealed that the full width at half height of the melting peak broadened with increasing ionic strength, indicating tighter water–protein binding at higher salt concentrations. Similarly, Zhang et al. [26] observed increased bound water content in fish meat with elevated salt levels. However, at 1.0 mol/L ionic strength, ΔH increased to 120.23 J/g. Under these circumstances, an abundance of Na^+^ and Cl^−^ disrupted the hydration layer of myosin via ion interaction [61,62].

### 3.5. Molecular Dynamics (MD) Simulations

Previous experimental results demonstrated that an ionic strength in the range of 0.3~0.8 mol/L could substantially enhance the solubility of myosin. In consideration of the practical scenario in surimi production [6], a comparative analysis was conducted between two systems: one with a conventional ionic strength of 0.5 mol/L and the other with a low ionic strength of 0.1 mol/L.

#### 3.5.1. Root Means Square Deviation (RMSD), Radius of Gyration (Rg)

**Figure 6:**
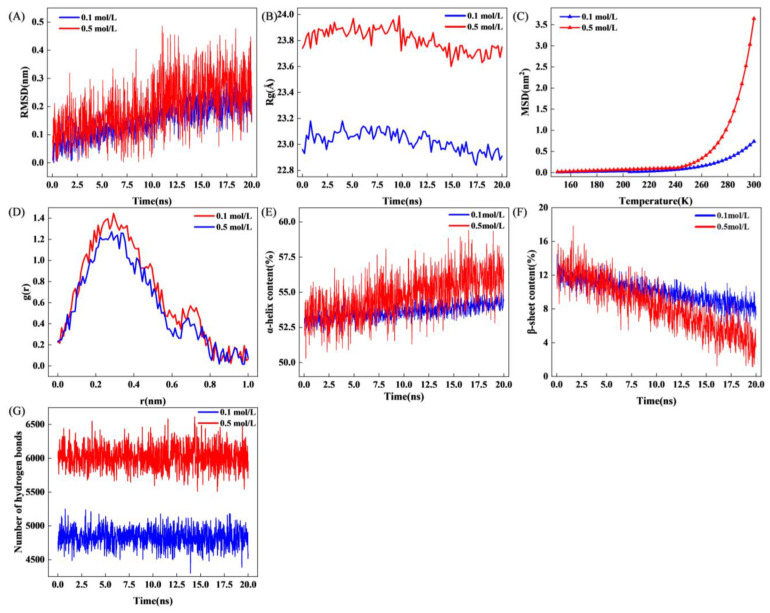
Molecular simulation results of myosin molecules at different ionic strengths. (**A**) root-mean square deviation; (**B**) molecular radius of rotation; (**C**) means displacement; (**D**) radial distribution function; (**E**) α-helix content; (**F**) β-sheet content; (**G**) number of hydrogen bonds.

RMSD is a key metric for monitoring time-dependent structural fluctuations and conformational stability of proteins. As shown in Figure 6A, the RMSD trajectories of myosin in 0.1 mol/L and 0.5 mol/L ionic strength systems gradually increased, with transient fluctuations, and leveled off after approximately 15 ns, indicative of progressive structural extension in the solvated environment. Throughout the entire simulation, the 0.5 mol/L system exhibited consistently higher RMSD values than the 0.1 mol/L system, implying that elevated ionic strength enhanced conformational flexibility and promoted structural expansion of myosin. These observations corroborated the UV (Figure 4C) and fluorescence (Figure 4E) results, confirming that ionic strength acted as an external trigger for myosin extending [29,63]. Figure 6B illustrates the radius of gyration (Rg) of myosin under two different ionic strength conditions. In the 0.1 mol/L system, Rg began at 23.0 Å and stabilized at approximately 22.9 ± 0.1 Å over time. In contrast, increasing the ionic strength to 0.5 mol/L raised the Rg to 23.7 Å, suggesting that an increase in ionic strength facilitated the extension of the myosin molecular structure, resulting in the relaxation of the protein conformation.

#### 3.5.2. Mean Squared Displacement (MSD)

In molecular dynamics simulations, protein molecules may undergo torsional transitions between different states due to increased temperature or changes in external conditions. This transition is reflected in the MSD, which exhibits a nonlinear increasing trend with alterations in simulation conditions [64]. Figure 6C depicted the impact of temperature changes on the MSD value of myosin. At 240 K, the MSD deviated from linearity and displayed a sudden increase, indicating that protein molecules underwent a “dynamic transition” during the molecular dynamic simulation. Liu et al. [65] highlighted that changes in the hydration shell precede and drove this transition, causing nonlinear increases in MSD near the critical temperature. In the results of this study, the growth trend of MSD values in the 0.5 mol/L ionic strength system was more pronounced compared to the 0.1 mol/L ionic strength system. This observation suggested that more hydrated water was bound within the myosin hydration layer in the former system [66].

#### 3.5.3. Radial Distribution Function (RDF)

The RDF characterizes the distribution of water molecules within and around protein molecules. “R” denotes the radial distance, describing the separation between the hydration layer and a reference point, while g (r) represents the particle density distribution, reflecting the variation in water molecule density at a distance “r” [67]. As illustrated in Figure 6D, the figure revealed the presence of two peaks for myosin under both ionic strength systems, indicating the existence of approximately two layers of water molecules on the protein surface. Notably, the 0.5 mol/L system exhibited higher g (r) values at both peaks compared to the 0.1 mol/L system, indicating a denser hydration shell. The increase in ionic strength promoted the extension of the myosin molecular structure, which substantially enhanced the attraction of the protein to water molecules, thereby facilitating the binding of more water molecules through hydrogen bonding. The increase in the relative density of water molecules near the protein ultimately manifested this effect. These results further verified that ionic strength could bind water molecules by inducing the exposure of polar groups, significantly increasing the thickness of the hydration layer [15].

#### 3.5.4. Changes in Secondary Structure During MD

Figure 6E,F tracked changes in secondary structure content over the simulation. In both systems, the α-helix content gradually increased with time. The increase was more pronounced in the 0.5 mol/L system, which ended with a higher α-helix fraction than the 0.1 mol/L system. Conversely, β-sheet content decreased accordingly. These results were consistent with the circular dichroism data (Figure 4F). Generally, the α-helix represents a relatively ordered and stable protein structure. As the ionic strength increased, the α-helix content gradually increased, indicating that the stability of myosin increased [11].

#### 3.5.5. Number of Hydrogen Bonds

Hydrogen bonds played a crucial role in maintaining protein structural stability and functional integrity. As shown in Figure 6G, the 0.5 mol/L system formed more myosin–water hydrogen bonds than the 0.1 mol/L system, confirming that increased ionic strength facilitated the establishment of a more compact, ordered hydration network. The increase in hydrogen bonding contributed to a denser and more orderly protein hydration layer, which aligned with the conclusions derived from the radial distribution function analysis (Figure 6D). Furthermore, hydrogen bonding was pivotal in maintaining the stability of protein secondary structures [39], and it supported the observation of a more stable myosin secondary structure under high ionic strength conditions.

#### 3.5.6. Conformation of Protein

**Figure 7:**
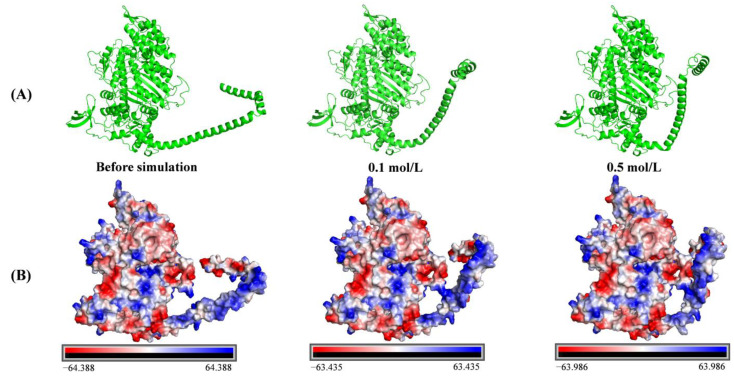
The 3D structural model and surface potential of myosin under different ionic strengths. (**A**) 3-D structural model; (**B**) surface potential.

The 3D structural changes in the myosin head region under different ionic strengths are shown in Figure 7A. Increasing the ionic strength led to a looser structure of myosin, indicating that elevated ionic strength induces exposure of amino acid residues in myosin. The exposure area increased with the increase in ionic strength, which is consistent with the tertiary structure results (Figure 4C). Figure 7B shows the changes in surface potential of the myosin head region under different ionic strengths. As ionic strength increased, the surface potential of myosin decreased and then increased, but the overall magnitude of change was relatively small. At low ionic strength, salt ions interacted with the charged residues on the protein surface, effectively neutralizing the charge and thereby reducing the surface potential. When the ionic strength increased, many ions were adsorbed on the protein, causing the surface potential of the protein to increase [46].

## 4. Discussion

This study investigated the effects of ionic strength on the solubility, spatial conformation, and hydration behavior of silver carp myosin through experimental measurements and molecular dynamics (MD) simulations. The findings provided new mechanistic insights into solubility behavior of myosin under varying ionic strength, which served as a theoretical guidance for improving surimi gel quality.

Increasing ionic strength from 0.0 to 0.6 mol/L significantly enhanced myosin solubility, while turbidity and average particle size decreased (Figure 2A–D). This was attributed to electrostatic shielding by Na^+^, which dissociated thick filaments into smaller oligomers or monomers, thereby increasing protein–water interactions. When ionic strength exceeded 0.8 mol/L, solubility plateaued and turbidity slightly increased, indicating a salting-out effect [30]. These observations were consistent with Gao et al. [17] and Xue et al. [29], who reported protein reaggregation at high salt concentrations. Concurrently, absolute Zeta potential value decreased (Figure 2E), confirming that Na^+^ neutralized surface carboxyl groups, weakened electrostatic interactions, and promoted more uniform protein distribution in the aqueous phase [33,34,35,36]. AFM images (Figure 3) further demonstrated that at moderate ionic strengths (0.3~0.6 mol/L), myosin formed uniformly small particles with good dispersion.

CD spectra (Figure 4A–B) revealed that the α-helix content increased from 46.8% to 60.2% with raising ionic strength from 0.0 to 0.8 mol/L, while β-sheet content decreased. Above 0.8 mol/L, α-helix content declined slightly and β-sheet content rebounded, reflecting disruption of the hydrogen-bond network and consequent loosening of the protein conformation. This structural change likely exposed more active groups, promoting protein–protein interactions and reaggregation [40,41]. UV spectra and fluorescence spectra (Figure 4C–E) further showed that moderate increases in ionic strength exposed more aromatic residues and hydrophilic groups, enhancing protein–water interactions. At an ionic strength of 1.0 mol/L, competition between ions and water induced partial refolding, reducing residue exposure [45,46,47]. This structural change might be a pivotal factor for the increase in turbidity (Figure 2B). Surface hydrophobicity (S_0_-ANS) rose within the ionic strength range of 0.0~0.6 mol/L and then plateaued (Figure 4F), which was consistent with the trends of solubility and microstructure. This further supported the notion that an increase in ionic strength promoted the extending of protein structures.

ITC (Figure 5A; Table 1) showed positive ΔH and ΔS alongside negative ΔG, suggesting that NaCl–myosin binding was driven primarily by hydrophobic interactions and was accompanied by order hydration-layer formation. Although the enthalpic response tended to decrease, no distinct stable state was observed within the tested range, suggesting that the binding process did not reach a classical thermodynamic saturation point under these conditions. This might be attributed to the continuous competition between Na^+^ and water molecules for the binding sites on the myosin surface, along with ongoing formation of the hydration layer [51,52,53,54,55]. BLI (Figure 5B) showed the strongest binding response in the ionic strength range of 0.2~0.4 mol/L, confirming maximal chain extension and hydration-layer thickening in that range. When the ionic strength exceeded 0.8 mol/L, the response tended to stabilize, reflecting competitive binding between ions and water molecules at protein surface sites under dynamic equilibrium. Thermal characteristics (Figure 5C,D; Table 2) revealed that increasing ionic strength led to a decrease in the peak temperature (T_p_), broadening of the peak width, and a decrease in ΔH, suggesting that more free water bound to myosin, thereby strengthening water–protein interactions [58,59,60]. When the ionic concentration was 1.0 mol/L, ΔH showed a slight increase. It was possible that excessive Na^+^ and Cl^−^ bound to the water molecules surrounding myosin via ionic interactions, thereby disrupted its hydration layer [61,62].

MD simulation results (Figure 6A–G) showed strong agreement with experimental findings. The 0.5 mol/L system exhibited higher RMSD and Rg values than that of 0.1 mol/L, indicating that elevated ionic strength promoted conformational extension of myosin. The high MSD values, high g (r) values, and elevated hydrogen bond numbers at this ionic strength suggested that the hydration layer became thicker and more ordered. A time-dependent increase in α-helix content further supported the CD results (Figure 4B).

**Figure 8:**
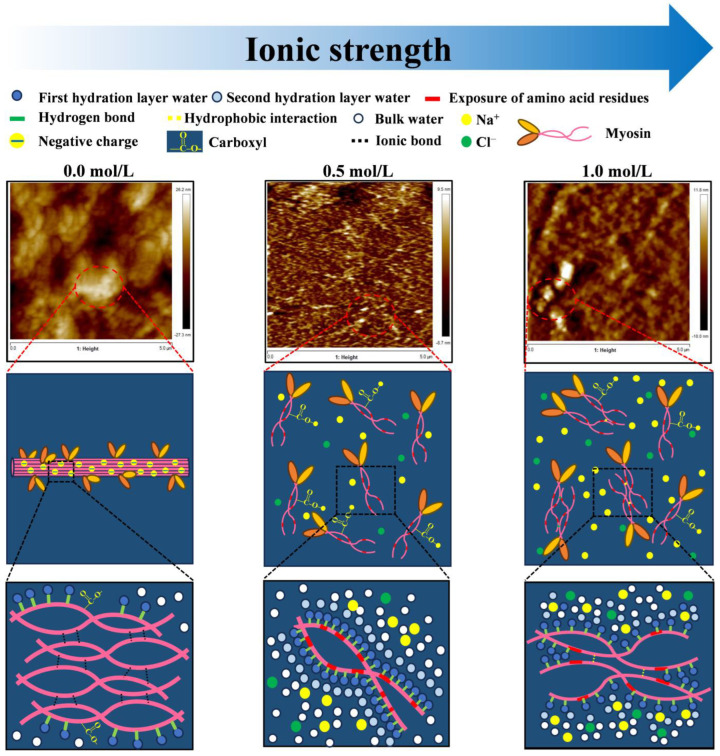
The mechanism underlying the effect of ionic strength on the spatial conformation and hydration of myosin.

Based on the above results, Figure 8 showed the mechanism underlying the effect of ionic strength on the spatial conformation and hydration of myosin. At low ionic strength (0.0~0.2 mol/L), myosin molecules assembled into thick filaments through electrostatic interactions. This aggregation process reduced solubility as aromatic residues embedded within the protein matrix, and polar groups were hindered from contacting water molecules, thereby diminishing hydration capacity. When ionic strength increased to 0.3~0.8 mol/L, Na^+^ bound to surface carboxyl groups, effectively shielding negative charges. This charge shielding induced the dissociation of thick filaments into monomers or oligomers, consequently improving solubility. At this stage, ionic strength promoted the formation of hydration layers by inducing exposure of polar groups to form more hydrogen bonds. However, when the ionic strength reached 1.0 mol/L, excessive Na^+^ competed with myosin for the surrounding water molecules, weakening the hydration layer. This weakening effect subsequently enhances hydrophobic interactions between molecules, ultimately leading to the formation of irregular aggregates.

## 5. Conclusions

This study elucidated the effects of varying ionic strengths on the relationship between myosin’s spatial conformation and hydration behavior. The results demonstrated that proper ionic strength (0.3~0.8 mol/L) significantly enhanced myosin solubility. This phenomenon was primarily attributed to the neutralization of negative surface charges on myosin molecules by the charge of ionic strength, which hindered protein aggregation. Moreover, this charge shielding effect promoted structural extension of myosin and exposed buried polar groups. These polar groups formed hydrogen bonds with water molecules, thereby augmenting hydration capacity. Consequently, proper ionic strength served as an effective regulator of myosin conformational expansion, hydration layer formation, and aggregation mitigation. In summary, this study revealed the intrinsic relationship between spatial conformation and hydration behavior of myosin as regulated by ionic strength, and its impact on solubility. These insights provide a theoretical basis for understanding how changes in ionic strength control the gel quality surimi products.

## Figures and Tables

**Table 1:** Thermodynamic parameters of myosin titrated by NaCl.

Sample	K (M^−1^ × 10^2^)	∆H (kJ·mol^−1^)	−T∆S (kJ·mol^−1^)	∆G (kJ·mol^−1^)
Myosin	3.87 ± 0.12	173.9 ± 1.8	−181.9 ± 2.1	−8.061 ± 0.504

**Table 2:** Effects of different ionic strengths on the thermal characteristics of myosin.

Ionic Strength (mol/L)	Onset Temperature T_o_ (°C)	Peak Temperature T_p_ (°C)	End Temperature T_e_ (°C)	∆H (J/g)
0.0	−7.65 ± 0.22 ^a^	0.77 ± 0.04 ^a^	3.27 ± 0.23 ^a^	262.97 ± 7.00 ^a^
0.2	−14.29 ± 2.88 ^b^	−0.70 ± 0.04 ^b^	1.65 ± 0.12 ^b^	213.67 ± 6.15 ^b^
0.4	−18.93 ± 0.15 ^c^	−1.94 ± 0.06 ^c^	0.29 ± 0.32 ^c^	188.50 ± 5.57 ^c^
0.6	−18.35 ± 1.01 ^c^	−3.20 ± 0.24 ^d^	−1.65 ± 0.36 ^d^	146.47 ± 8.84 ^d^
0.8	−19.14 ± 1.16 ^c^	−4.96 ± 0.34 ^e^	−4.31 ± 0.34 ^e^	110.85 ± 7.89 ^e^
1.0	−21.68 ± 0.22 ^d^	−5.93 ± 0.20 ^f^	−5.02 ± 0.22 ^f^	120.23 ± 8.58 ^e^

Note: Different lowercase letters indicate significant difference between different ionic strengths (*p* < 0.05).

## Data Availability

The original contributions presented in the study are included in the article, further inquiries can be directed to the corresponding author.
